# Innovative herbal oil formulation as a pre-prosthetic mucosal conditioner: a prospective controlled cytological study on the degree of keratinization in edentulous ridge mucosa before and after Its topical application

**DOI:** 10.3389/fdmed.2026.1903095

**Published:** 2026-08-26

**Authors:** Savitha Dandekery, Rajesh Shetty, Sanath Shetty, Suman B, Mallikarjuna Ragher, Riaz Abdulla, Uma M. Prabhu

**Affiliations:** 1 Department of Prosthodontics, Yenepoya Dental College, Yenepoya University, Mangalore, India; 2 Department of Public Health Dentistry, KVG Dental College and Hospital, Sullia, India

**Keywords:** cytocompatibility, edentulous ridge, exfoliative cytology, herbal dentistry, keratinization, moringa oleifera (MO), mucosal conditioning, prosthodontics

## Abstract

**Background:**

Adequate keratinization of the edentulous ridge is fundamental for denture support, retention, stability, and mucosal resilience. This study evaluated the effect of topical Moringa oleifera stem bark oil on oral mucosal keratinization in edentulous ridges using a prospective controlled clinical trial design with a water-massage control group.

**Methods:**

One hundred and eight completely edentulous participants were enrolled between April 2025 and September 2025: a treatment group (*n* = 54) received twice-daily topical Moringa oleifera stem bark oil for four weeks, and a control group (*n* = 54) received twice-daily water massage of the same ridge area. Exfoliative cytology was performed at baseline (T0), Week 2 (T2), and Week 4 (T4). The *in vitro* cytocompatibility of the oil was additionally evaluated in human keratinocyte (HaCaT) cells using the MTT assay, performed in three independent experiments (biological triplicate), each with three technical replicates per concentration. Statistical analyses included the Friedman test (within-group temporal changes), the Wilcoxon signed-rank test with Bonferroni correction (post-hoc pairwise comparisons), and the Mann–Whitney U test (between-group comparisons) for the clinical cytology data. Effect sizes are reported as Kendall's W and rank-biserial correlation (r).

**Results:**

The treatment group demonstrated a significant progressive increase in superficial keratinized cells, from a median of 57.5 at T0 to 114.0 at T4 [Friedman *χ*^2^(2) = 59.40, *p* < 0.001, Kendall's W = 0.632]. *Post-hoc* Wilcoxon analysis confirmed significant improvements at each interval (all Bonferroni-corrected *p* < 0.001, r = 0.615–0.796). The control group showed no significant temporal change in superficial cells (Friedman *p* = 0.162, Kendall's W = 0.037). Between-group differences became more pronounced over time: superficial cell counts were not significantly different at baseline (*p* = 0.433), diverged at Week 2 (*p* = 0.002, r = 0.319), and were highly significantly different at Week 4 (*p* < 0.001, r = 0.777). The MTT assay (three independent experiments, each with technical triplicates; *n* = 3 per concentration) showed that the oil was non-toxic to HaCaT keratinocytes at concentrations up to 50 μg/mL. Mean cell viability trended higher than the untreated control in a concentration-dependent manner; however, this comparison was descriptive only, as no inferential statistical test was applied to the *in vitro* dataset, and the apparent trend should not be interpreted as a statistically confirmed effect. No adverse reactions were observed clinically.

**Conclusion:**

Topical Moringa oleifera stem bark oil was associated with a significant increase in cytological markers of keratinization, with no change in the water-massage control group, and descriptively demonstrated favourable *in vitro* cytocompatibility, with viability trending higher than control across tested concentrations (mean ± SD, three independent experiments); this *in vitro* trend was not evaluated by inferential statistics and warrants confirmation with formal statistical comparison in future work. These findings suggest promising potential as a pre-prosthetic mucosal conditioning approach, pending confirmation in larger, randomized, placebo (vehicle)-controlled trials with longer follow-up, histological verification, and patient-centred outcomes. Clinical Trial Registration: CTRI/2025/04/084650; Patent Publication No. 202241052361.

**Clinical Trial Registration:**

(https://ctri.nic.in) CTRI/2025/04/084650; Patent Publication No. 202241052361.

## Introduction

Successful removable prosthetic rehabilitation depends critically on the quality of the denture-bearing mucosa. The oral mucosa overlying the edentulous ridge must possess adequate keratinization to withstand the mechanical forces of a denture base and resist microbial irritation (1). Clinically, keratinized mucosa provides a resilient, compressible, yet durable surface essential for denture support, stability, and retention; the significance of adequate keratinization extends beyond removable prosthetics and has been well established in implant-supported prosthetics as well (2, 3). When keratinization is deficient, patients experience mucosal soreness, persistent ulceration, chronic inflammation, and poor denture tolerance (4, 5).

The epidemiology of edentulism is a growing public health concern. Globally, approximately 23% of individuals above the age of 60 are completely edentulous, and with advancing age the oral mucosa undergoes predictable histological changes—it becomes thinner, less hydrated, and more susceptible to trauma (6). After tooth extraction, residual ridge resorption progressively reduces the available alveolar foundation while the overlying mucosal tissues lose their keratinized character, rendering them less able to provide adequate prosthetic support (7).

Several strategies have been explored to enhance mucosal keratinization. Radke et al. demonstrated significant increases in superficial keratinized cells following a four-week astringent massage protocol in edentulous patients (8). Similarly, Menon et al. confirmed the efficacy of an astringent gel in improving palatal mucosal keratinization (9). Beyond astringents, other plant-derived agents such as Aloe vera gel and propolis have also been investigated for denture-related mucosal management, with generally favourable but limited evidence (10, 11). These studies collectively highlighted limitations of existing conditioning agents, including irritative potential and, in several instances, the absence of a concurrent control arm capable of distinguishing the pharmacological effect of the agent from the non-specific effect of mucosal massage (12).

Moringa oleifera Lam.—widely known as the “miracle tree”—has attracted significant scientific interest owing to its antioxidant, anti-inflammatory, keratinocyte-protective, and wound-healing properties (13–15). Its bioactive constituents, including flavonoids, tocopherols, sphingosine-1-phosphate (S1P), sesamin, and gallic acid, promote cell proliferation, accelerate wound closure, and protect keratinocytes against oxidative damage (16, 17). Most prior investigations of Moringa oleifera in oral health have used aqueous leaf extracts (13, 18–20). In the present study, stem bark oil was selected instead, prepared according to classical Ayurvedic taila-paka (oil-processing) principles (21), on the rationale that its lipophilic character may allow more efficient partitioning into, and retention within, the lipid-rich mucosal epithelium compared with aqueous leaf-based preparations; this hypothesis, however, had not previously been tested in a controlled clinical setting. The use of Moringa stem bark oil specifically for augmenting edentulous ridge keratinization has not, to our knowledge, been systematically investigated using a controlled clinical trial design.

The present study addresses this gap by employing a prospective controlled clinical trial design with a concurrent water-massage control group, compliant with the Consolidated Standards of Reporting Trials (CONSORT). This design enables an assessment of whether keratinization changes in the treatment group exceed changes attributable to the mechanical effect of massage alone, helping to distinguish the contribution of the Moringa formulation from the non-specific effect of mucosal massage. The primary objective was to evaluate and compare the degree of keratinization in edentulous ridges at baseline, two weeks, and four weeks in both treatment and control participants. A secondary objective was to evaluate the *in vitro* cytocompatibility of the Moringa oleifera stem bark oil in human keratinocytes (HaCaT cells) using the 3-(4,5-dimethylthiazol-2-yl)-2,5-diphenyltetrazolium bromide (MTT) assay.

## Materials and methods

### Study design and ethical approval

This prospective, controlled, exploratory clinical trial was conducted at the Department of Prosthodontics, Yenepoya Dental College, Yenepoya (Deemed to be University), Mangalore, India. Institutional Ethics Committee approval was obtained (YEC-1/2023/329). All participants provided written informed consent in accordance with the Declaration of Helsinki. Clinical trial registration: CTRI/2025/04/084650 (https://ctri.nic.in); Patent Publication No. 202241052361.

**Figure 1:**
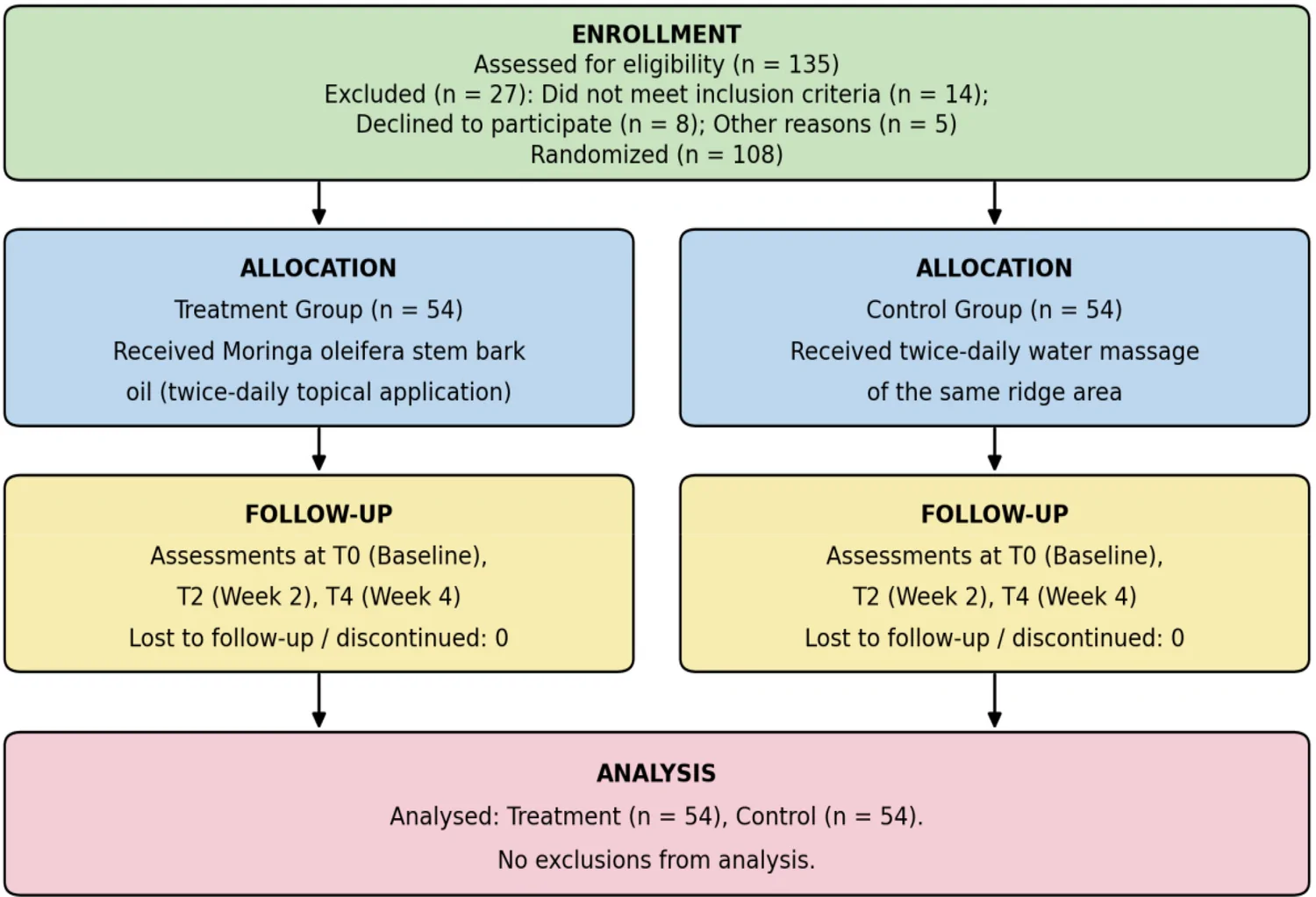
CONSORT flow diagram showing participant enrollment, allocation, follow-up, and analysis.

The study was conducted and reported in accordance with the CONSORT guidelines. A CONSORT flow diagram is provided in Figure 1.

### Participants

Participant enrolment commenced on 25 April 2025 and was completed on 14 November 2025. The intervention period concluded on 28 May 2025, and all follow-up assessments were completed by 28 June 2025.

A total of 108 completely edentulous participants aged 50–70 years were enrolled and allocated into two groups: a treatment group (*n* = 54) receiving Moringa oleifera stem bark oil, and a control group (*n* = 54) receiving twice-daily water massage of the right posterior mandibular edentulous ridge. The two groups were comparable at baseline in age, sex, edentulous duration, and body mass index (BMI) (all *p* > 0.10; Table 1).

**Table 1:** Participant demographic and baseline characteristics.

Variable	Treatment Group (*n* = 54)	Control Group (*n* = 54)	*p*-value
Age (years), Mean ± SD	61.2 ± 5.8	62.1 ± 6.0	0.498
Sex (Male/Female)	30/24	29/25	0.843
Edentulous duration (years), Mean ± SD	5.7 ± 3.4	5.9 ± 3.5	0.783
BMI (kg/m^2^), Mean ± SD	24.6 ± 3.6	24.8 ± 3.7	0.812
Intervention	Moringa oil (twice daily × 4 weeks)	Water massage (twice daily × 4 weeks)	—

BMI, body mass index; SD, standard deviation.

Inclusion criteria: age 50–70 years, complete edentulism, requiring removable prosthetic rehabilitation. Exclusion criteria: active tobacco or betel nut use, neuromuscular disorders, submucosal fibrosis, recent dental extractions (within three months), visible ridge inflammation, systemic disease, and unwillingness to adhere to the study protocol.

### Randomization and allocation concealment

The random allocation sequence was computer-generated using block randomization with stratification. Allocation concealment was ensured through the use of sequentially numbered, opaque, sealed envelopes (SNOSE), which were opened only at the time of participant enrolment.

### Sample size justification

An *a priori* power calculation was performed to justify recruitment of 54 participants per group for the clinical cytology comparisons, based on an assumed effect size of 0.05, a two-sided significance level (alpha) of 0.05, and a statistical power of 0.84. This power calculation applies to the human clinical cohort only and is distinct from the *in vitro* replicate structure of the MTT assay described below.

### Moringa oleifera Oil formulation and application protocol

The Moringa oleifera stem bark oil was formulated in a Good Manufacturing Practice (GMP)-certified laboratory using a solvent-extraction-based method, consistent with classical Ayurvedic taila-paka processing principles (21).

Physicochemical characterization confirmed: pH 6.74, acid value 2.20 mg KOH/g, iodine value 87.81 g I₂/100 g, saponification value 195.24, and moisture content 0.15%.

Quantitative batch-to-batch reproducibility data across multiple production batches, and further quantitative phytochemical characterization of the major bioactive constituents (e.g., by HPLC or LC-MS), were not performed for this study and are acknowledged as a limitation.

Each treatment group participant applied one drop (0.05 mL) of oil to the right posterior mandibular edentulous ridge twice daily for four weeks, followed by gentle massage with the index finger for five minutes. Control group participants applied one drop of water to the same ridge site and performed an identical five-minute gentle massage with the index finger twice daily for four weeks.

### Cytocompatibility assay (MTT assay)

The cytocompatibility of the Moringa oleifera stem bark oil was evaluated *in vitro* using human keratinocyte (HaCaT) cells and the MTT [3-(4,5-dimethylthiazol-2-yl)-2,5-diphenyltetrazolium bromide] assay (22). HaCaT cells were inoculated into 96-well microtiter plates containing complete culture medium and incubated at 37 °C in a humidified atmosphere of 5% CO₂. Cells were treated with Moringa oleifera stem bark oil at concentrations of 0 (untreated control), 5, 10, 25, and 50 μg/mL and incubated for 24 h. MTT reagent was then added to each well, and the plates were incubated at 37 °C for a further 4 h. The resulting formazan crystals were dissolved in dimethyl sulfoxide (DMSO), and optical density was measured at 570 nm using a multimode microplate reader. Percentage cell viability was calculated relative to the untreated control (set at 100%).

Each concentration was tested in three technical replicate wells per plate, and the entire experiment was independently repeated on three separate occasions (*n* = 3 independent biological experiments per concentration; 9 individual well readings per concentration in total). Results are reported descriptively as mean percentage cell viability ± standard deviation (SD) across the three independent experiments. No inferential statistical test (e.g., one-way ANOVA or Kruskal–Wallis test) was applied to compare viability across concentrations, and normality/variance assumptions were therefore not formally assessed for this dataset. Accordingly, the MTT findings should be interpreted as descriptive and exploratory rather than as a statistically confirmed comparison between concentrations; this is discussed further as a limitation below.

### Exfoliative cytology

Exfoliative cytology samples were collected from the right posterior mandibular edentulous ridge at three time points: baseline (T0), Week 2 (T2), and Week 4 (T4). Smears were fixed in 95% ethyl alcohol, stained using the Papanicolaou technique (23), and viewed under low-power magnification (10 × ). One hundred cells per slide were classified as superficial or intermediate according to established Papanicolaou criteria. Cytological evaluation was performed by a single trained examiner blinded to group allocation. Intraclass correlation coefficients (ICC) were ≥0.80. Intra-observer reproducibility was reassessed at each of the three study time points (T0, T2, and T4), rather than only during initial calibration.

**Figure 2:**
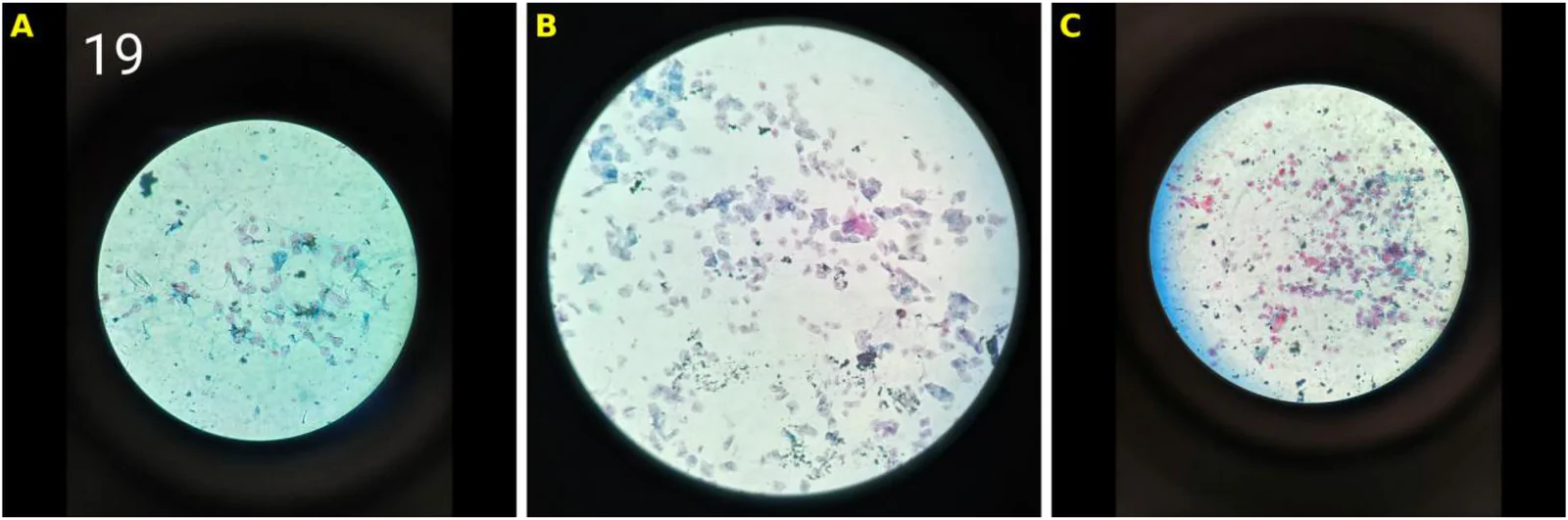
Representative photomicrographs of papanicolaou-stained exfoliative cytology smears from the treatment group, viewed under low-power magnification (10×), at **(A)** baseline (T0), **(B)** week 2 (T2), and **(C)** week 4 (T4), illustrating the progressive increase in superficial (keratinized) squamous cells over the treatment period.

Representative photomicrographs of cytological smears at T0 (Figure 2A), T2 (Figure 2B), and T4 (Figure 2C) are presented in Figure 2.

### Statistical analysis

All statistical analyses were performed using SPSS Statistics version 26.0 (IBM Corp., Armonk, NY, USA). Non-parametric methods were employed throughout for the clinical cytology data, given the non-normal distribution of cytological cell counts confirmed by the Shapiro–Wilk test. Within-group temporal changes were evaluated using the Friedman test; effect sizes are reported as Kendall's W (small ≥0.1, medium ≥0.3, large ≥0.5). *Post-hoc* pairwise comparisons were performed using the Wilcoxon signed-rank test with Bonferroni correction (*α*-adjusted = 0.017). Between-group comparisons were performed using the Mann–Whitney U test; effect sizes are reported as rank-biserial correlation r (small ≥0.1, medium ≥0.3, large ≥0.5).

Cytocompatibility (MTT assay) data are reported descriptively as mean percentage cell viability ± SD relative to the untreated control, based on three independent experiments performed in technical triplicate; no inferential statistical test was applied to this dataset (see Cytocompatibility Assay section above). A *p*-value < 0.05 was considered statistically significant for the clinical cytology analyses described above.

Treatment and control groups were confirmed to be comparable at baseline for age, sex, edentulous duration, and BMI (Table 1); however, formal covariate-adjusted modelling (e.g., ANCOVA or regression-based analysis) incorporating age, sex, BMI, and diabetic status was not performed in the primary analysis, as the participant-level dataset required for this analysis was not available — this is acknowledged as a limitation below.

Confidence intervals were likewise not computed for the effect-size estimates below, as the underlying participant-level dataset required to compute them was not accessible for the present analysis; this is also acknowledged as a limitation.

## Results

One hundred and eight participants completed the study without dropouts or adverse reactions. Baseline characteristics were comparable between groups (Table 1). No statistically significant between-group differences were observed in age (*p* = 0.498), sex distribution (*p* = 0.843), edentulous duration (*p* = 0.783), or BMI (*p* = 0.812), confirming successful group comparability.

Descriptive statistics of superficial and intermediate cell counts across all time points for both groups are summarized in Table 2. The treatment group demonstrated a marked progressive increase in superficial cell counts, with mean values rising from 61.2 ± 27.7 at baseline to 82.1 ± 31.4 at Week 2 and 118.8 ± 37.0 at Week 4. Intermediate cell proportions declined correspondingly. In contrast, the control group (water massage) showed temporal stability in cell-type proportions across all three time points, with superficial cell means of 62.7 ± 6.4, 63.8 ± 6.2, and 64.0 ± 6.9 at T0, T2, and T4, respectively, consistent with the absence of a marked keratinization change attributable to massage alone.

**Table 2:** Descriptive statistics of epithelial cell types across time points for both groups (mean ± SD; median, IQR).

Group	Cell Type	Baseline (T0) Mean ± SD (Median, IQR)	Week 2 (T2) Mean ± SD (Median, IQR)	Week 4 (T4) Mean ± SD (Median, IQR)
Treatment (*n* = 54)	Superficial	61.2 ± 27.7 (57.5, 39.8)	82.1 ± 31.4 (78.5, 42.2)	118.8 ± 37.0 (114.0, 49.0)
	Intermediate	28.1 ± 19.7 (27.0, 27.8)	22.0 ± 16.6 (19.0, 18.5)	20.6 ± 19.4 (14.0, 20.0)
Control (*n* = 54)	Superficial	62.7 ± 6.4 (64.0, 11.0)	63.8 ± 6.2 (63.0, 9.0)	64.0 ± 6.9 (64.0, 11.0)
	Intermediate	35.9 ± 6.9 (36.0, 7.0)	34.5 ± 5.5 (34.0, 4.0)	34.2 ± 6.7 (35.0, 8.0)

IQR, interquartile range. Cell counts represent absolute counts per 100-cell cytological smear evaluation.

Between-group comparisons of superficial and intermediate cell proportions at each time point are presented in Table 3. At baseline, superficial cell counts were not significantly different between the treatment and control groups (U = 1,329, Z = −0.79, *p* = 0.433, r = 0.080), consistent with pre-intervention group equivalence. The groups diverged at Week 2, with the treatment group demonstrating substantially higher superficial cell counts than the control group (U = 1,969, Z =  + 3.14, *p* = 0.002, r = 0.319; medium–large effect). By Week 4, the between-group difference was more pronounced (U = 2,283, Z =  + 7.69, *p* < 0.001, r = 0.777; large effect), with the treatment group median (114.0) nearly double that of the control group (64.0).

**Table 3:** Between-group comparisons of epithelial cell proportions (Mann–Whitney U test).

Time Point	Cell Type	Treatment Median (IQR)	Control Median (IQR)	U	Z	*p*-value/r
Baseline (T0)	Superficial	57.5 (39.8)	64.0 (11.0)	1,329	−0.79	0.433/r = 0.080 (ns)
	Intermediate	27.0 (27.8)	36.0 (7.0)	808	−2.66	0.008/r = 0.270 (**)
Week 2 (T2)	Superficial	78.5 (42.2)	63.0 (9.0)	1,969	+3.14	0.002/r = 0.319 (**)
	Intermediate	19.0 (18.5)	34.0 (4.0)	466	−5.13	<0.001/r = 0.521 (***)
Week 4 (T4)	Superficial	114.0 (49.0)	64.0 (11.0)	2,283	+7.69	<0.001/r = 0.777 (***)
	Intermediate	14.0 (20.0)	35.0 (8.0)	429	−5.48	<0.001/r = 0.554 (***)

r, rank-biserial correlation (small ≥0.1, medium ≥0.3, large ≥0.5). ** *p* < 0.01, *** *p* < 0.001, ns, not significant.

Within-group temporal analysis using the Friedman test is presented in Table 4. In the treatment group, superficial cell counts showed a highly significant temporal change across the three measurement points [*χ*^2^ (2) = 59.40, *p* < 0.001, Kendall's W = 0.632; large effect]. Intermediate cell counts also showed a significant temporal decline [*χ*^2^(2) = 14.72, *p* < 0.001, W = 0.157; small effect]. In contrast, the control group demonstrated no significant temporal change in superficial cell counts [*χ*^2^(2) = 3.64, *p* = 0.162, W = 0.037], nor in intermediate cell counts after Bonferroni adjustment [*χ*^2^(2) = 8.01, *p* = 0.018, W = 0.082; negligible effect]. This pattern is consistent with the keratinization change observed in the treatment group reflecting a response associated with the Moringa oil intervention, rather than a temporal or massage-related effect alone; as discussed below, however, this comparison does not by itself rule out all non-specific vehicle or contact effects.

**Table 4:** Within-group temporal changes in epithelial cell proportions (friedman test).

Group	Cell Type	*χ*^2^ (2)	*p*-value	Kendall's W	Effect Size Interpretation
Treatment	Superficial	59.40	<0.001	0.632	Large; significant progressive increase
	Intermediate	14.72	<0.001	0.157	Small; significant progressive decrease
Control	Superficial	3.64	0.162	0.037	Negligible; no significant change
	Intermediate	8.01	0.018	0.082	Negligible; ns after Bonferroni adjustment

W, Kendall's W coefficient of concordance. Effect size thresholds: negligible <0.10, small ≥0.10, medium ≥0.30, large ≥0.50. ns, not significant.

Post-hoc Wilcoxon signed-rank test results with Bonferroni correction are presented in Table 5. For the treatment group, superficial keratinized cell improvements were significant at every interval: T0 vs. T2 [W = 217.5, p(Bonferroni) = 0.0007, r = 0.615], T2 vs. T4 (W = 80.5, *p* < 0.001, r = 0.746), and T0 vs. T4 (W = 48.0, *p* < 0.001, r = 0.796), with effect sizes increasing across successive intervals. For intermediate cells, only the T0-vs.-T4 comparison reached Bonferroni-adjusted significance (W = 300.0, *p* = 0.026, r = 0.408; medium effect). For the control group, no pairwise comparison for superficial cells at any interval reached significance (all *p* > 0.09). Confidence intervals for these effect-size estimates were not calculated in the original analysis and are recommended for a future re-analysis of the underlying dataset.

**Table 5:** *Post-Hoc* pairwise comparisons of epithelial cell proportions (Wilcoxon signed-rank test with Bonferroni correction).

Group	Cell Type	Comparison	*p* (raw)	*p* (Bonferroni)	W	Effect Size (r)
Treatment	Superficial	T0 vs. T2	0.0002	0.0007 ***	217.5	0.615 (large)
		T2 vs. T4	<0.001	<0.001 ***	80.5	0.746 (large)
		T0 vs. T4	<0.001	<0.001 ***	48.0	0.796 (large)
	Intermediate	T0 vs. T2	0.080	0.241 ns	380.5	0.255 (small)
		T2 vs. T4	0.177	0.531 ns	436.5	0.197 (small)
		T0 vs. T4	0.0086	0.026 *	300.0	0.408 (medium)
Control	Superficial	T0 vs. T2	0.205 ns	—	425.0	0.266
		T2 vs. T4	0.730 ns	—	465.5	0.209
		T0 vs. T4	0.092 ns	—	405.0	0.295

r, rank-biserial correlation effect size. * *p* < 0.05, *** *p* < 0.001, ns, not significant. Bonferroni-adjusted α = 0.017.

**Figure 3:**
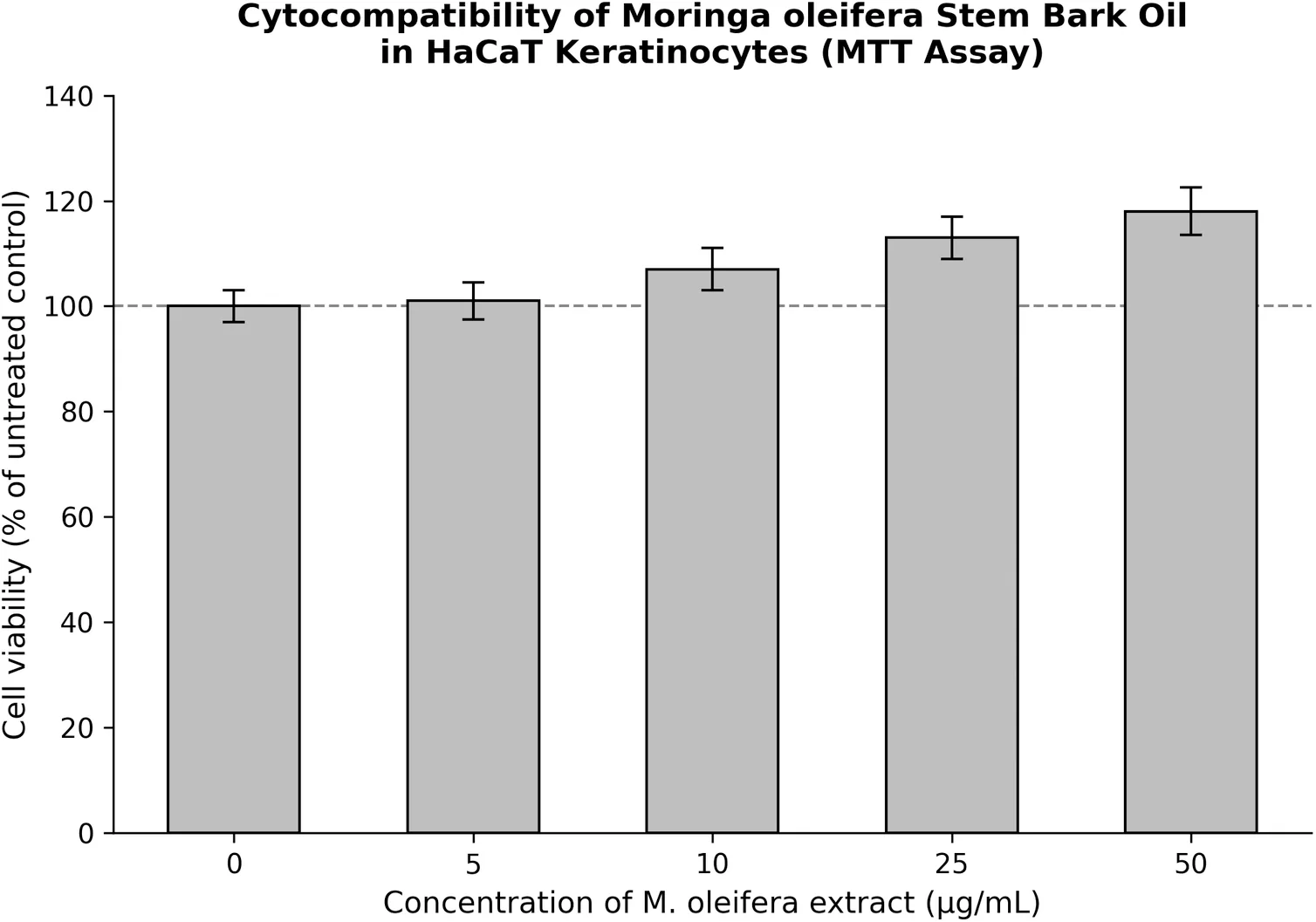
Cytocompatibility of the moringa oleifera stem bark oil in haCaT keratinocytes (MTT assay). Data are presented as mean percentage cell viability relative to the untreated control (0 μg/mL, set at 100%), ± standard deviation (SD), derived from three independent experiments (*n* = 3), each performed in technical triplicate. No inferential statistical comparison between concentrations was performed; the apparent concentration-dependent increase in viability is descriptive only.

The MTT assay (three independent experiments, each with three technical replicates; *n* = 3 per concentration) demonstrated that Moringa oleifera stem bark oil was non-toxic to HaCaT keratinocytes at all concentrations tested, up to 50 μg/mL. Mean percentage cell viability (± SD) relative to the untreated control (100 ± 4.8%) was 101.2 ± 5.1% at 5 μg/mL, 107.0 ± 4.5% at 10 μg/mL, 113.2 ± 4.2% at 25 μg/mL, and 118.2 ± 5.6% at 50 μg/mL, showing a concentration-dependent upward trend in viability relative to control. As no inferential statistical test was performed on this dataset, this trend is reported descriptively and should not be interpreted as a statistically confirmed increase in cell proliferation; it is presented as supportive, hypothesis-generating evidence of favourable cytocompatibility, consistent with the absence of any cytotoxic signal across the tested concentration range (Figure 3).

## Discussion

The primary methodological advance of the present study over prior work in this area is the inclusion of a concurrent water-massage control group within a registered clinical trial framework. Previous investigations of non-surgical mucosal conditioning—including the studies of Radke et al. and Menon et al.—employed single-arm pre–post designs, which cannot readily distinguish the pharmacological effect of the conditioning agent from natural temporal variation, the Hawthorne effect, or the non-specific mechanical effect of mucosal massage (8, 9). In the present data, the control group, assessed at identical time points with water massage alone, showed no significant change in superficial cell counts across four weeks (Friedman *p* = 0.162, W = 0.037). This finding suggests that massage alone is unlikely to produce a keratinization change of comparable magnitude within a four-week period and lends support to—though does not by itself definitively confirm—a pharmacological contribution of the Moringa oleifera oil to the changes observed in the treatment group; a vehicle (inert-oil) control arm, discussed further below, would be required to fully isolate this effect.

The treatment group demonstrated a progressive and accelerating keratinization change across the study period. Superficial cell medians increased from 57.5 at T0 to 78.5 at T2 (+36%) and further to 114.0 at T4 (+45% from T2; +98% from T0), with the magnitude of change accelerating in the second two-week interval. This pattern may reflect cumulative loading of bioactive constituents within the mucosal epithelium, with initial weeks establishing pharmacokinetic equilibrium and subsequent weeks producing a larger biological effect; this remains a hypothesis to be tested rather than a demonstrated mechanism. The effect size at the final time point (r = 0.777 in the between-group comparison; r = 0.796 in the T0-vs.-T4 within-group comparison) indicates a statistically robust change, although its clinical significance—in terms of denture comfort, retention, or patient-reported outcomes—was not directly assessed in this study and should not be assumed. Nonetheless, participants were monitored at every follow-up visit for denture-associated ulceration and mucosal pain, and no such adverse findings were documented in either group throughout the study period, providing preliminary reassurance regarding the tolerability of the formulation.

The biological plausibility of the observed keratinization change is supported by the reported phytochemical composition of the formulation, including tocopherol, gallic acid, sesamin, and S1P. Tocopherol has established roles in keratinocyte membrane protection and reduction of oxidative stress-mediated apoptosis (24). Gallic acid has been reported to accelerate keratinocyte migration and proliferation through activation of focal adhesion kinase (FAK), c-Jun N-terminal kinase (JNK), and extracellular signal-regulated kinase (ERK) signalling pathways in prior *in vitro* and mechanistic studies (25, 26). Sesamin-containing Moringa preparations have demonstrated anti-inflammatory and wound-healing effects in other experimental models (27, 28), and S1P is an established regulator of keratinocyte differentiation and epidermal homeostasis (29). Zhou et al. demonstrated that Moringa stem extract protects HaCaT keratinocytes against oxidative stress through enhancement of antioxidant defence enzymes and activation of the Nrf2 pathway (29), and hydrogel preparations incorporating Moringa leaf extract have shown enhanced wound healing in diabetic tissue models (30). Consistent with these literature-based mechanistic findings, the *in vitro* MTT assay performed in the present study directly confirmed that the study formulation itself was non-toxic to HaCaT keratinocytes at concentrations up to 50 μg/mL and showed a descriptive, concentration-dependent trend toward higher viability relative to control across three independent experiments, providing directional supportive evidence for the keratinocyte-compatible properties of the specific oil used in this trial; because this comparison was not evaluated by inferential statistics, it should be regarded as hypothesis-generating rather than as confirmed proof of a proliferative effect, and it complements—rather than independently establishes—the mechanistic inferences drawn from other Moringa preparations reported in the literature (22).

Other proposed molecular mechanisms underlying the observed clinical effect, however, remain inferred from prior *in vitro* and animal studies rather than directly demonstrated in this trial and should be regarded as a proposed rather than a confirmed explanation for the cytological changes observed here.

The present findings are broadly consistent with, and extend, prior single-arm investigations of Moringa in dentistry and mucosal conditioning. Radke et al. reported mean superficial cell proportions increasing from 79.8% to 90.6% over four weeks with astringent massage (8); the present treatment group demonstrated a comparable or numerically greater relative increase, while the water-massage control group did not show an equivalent change—consistent with, though not proof of, a specific pharmacological contribution from the Moringa formulation. Beyond astringents, other plant-derived agents have also been evaluated for denture-related mucosal management: Aloe vera gel has shown benefit as a mucosal conditioning agent in complete denture wearers in a randomized controlled trial (10), and propolis gel has shown efficacy in the management of denture stomatitis in a pilot study (11), although evidence for both remains limited in scope. Buakaew et al. demonstrated significant reductions in plaque and gingival indices with Moringa-containing mouthwash compared with control (18); Duarte et al. demonstrated antiplaque efficacy of a Moringa oleifera-containing dentifrice in a randomized clinical setting (19); and Lingawi reported promising outcomes for Moringa in caries prevention and enamel remineralization (20). Taken together, this body of evidence—spanning periodontal, mucosal, and prosthetic applications, and including comparator agents such as Aloe vera and propolis—supports further investigation of Moringa formulations in clinical dentistry, though direct head-to-head comparisons with these alternative agents have not yet been performed.

An important distinction of the present study is the use of stem bark oil rather than the more widely investigated leaf extract. The stem bark oil formulation, prepared according to classical Ayurvedic taila-paka principles, may contain a distinct phytochemical profile enriched with lipophilic constituents that could plausibly confer different mucosal penetration and tissue-level retention characteristics compared with aqueous leaf preparations; however, this comparison was not directly tested in the present trial, and quantitative phytochemical characterization of the formulation (for example, by HPLC or LC-MS) was not performed, which limits the strength of this comparison.

Pre-prosthetic mucosal conditioning is a recognized but underutilized strategy, in part because few non-invasive pharmacological agents have been evaluated in controlled clinical trials. Moringa oleifera has also shown therapeutic effects on tongue mucosal tissue in animal models (31) and osseointegration-enhancing properties in implant models (32), suggesting potential relevance across a broader range of prosthetic applications. However, because the present study evaluated only a cytological surrogate marker over a relatively short period, these clinical implications should be regarded as preliminary; immediate clinical adoption is not supported by the current evidence, and the findings should instead motivate further trials incorporating clinically meaningful, patient-centred endpoints before any practice recommendation is made (36, 37).

### Limitations

This study has several limitations that should be considered when interpreting the findings. Although a water-massage control group was used to account for the non-specific mechanical effect of massage, the control did not receive an inert oil vehicle, so the specific pharmacological contribution of the Moringa formulation cannot be fully separated from potential effects of an oil-based carrier. Participants and those administering the intervention were not blinded to group allocation, although cytological scoring itself was performed by an examiner blinded to allocation. The study was conducted at a single centre, which may limit generalizability. Keratinization was assessed only by exfoliative cytology, an indirect and minimally invasive surrogate marker; true epithelial keratinization was not confirmed histologically, and this should be considered when interpreting the cytological findings as evidence of a keratinization change. The four-week follow-up period is relatively short for evaluating pre-prosthetic tissue conditioning, and it remains unknown whether the observed cytological changes are sustained, reversible, or transient after treatment cessation; longer follow-up, including assessment after discontinuation of the intervention, is needed. Clinically meaningful, patient-centred outcomes—including denture comfort, prosthesis retention and stability, and oral health-related quality of life—were not systematically assessed using validated instruments, and no data on long-term prosthetic performance are available. We acknowledge that these broader patient-centred outcomes were not formally assessed in the present study; nevertheless, participants were evaluated at each follow-up visit for the presence of denture-associated ulcerations and mucosal pain, and these parameters were documented as part of the clinical follow-up assessment. Quantitative phytochemical characterization of the major bioactive constituents (for example, by HPLC or LC-MS) was not performed; only physicochemical parameters and *in vitro* cytocompatibility were assessed, and batch-to-batch reproducibility data are not presented in this analysis. For the MTT assay specifically, although the experiment was performed in biological triplicate (three independent experiments) with technical triplicates per concentration, no inferential statistical test (e.g., one-way ANOVA with *post-hoc* correction, or a non-parametric equivalent) was applied to formally compare viability across concentrations, and normality and variance assumptions were consequently not tested for this dataset; the reported viability trend is therefore descriptive and exploratory, and future studies should apply formal inferential statistics, with assumptions verified, to confirm whether the observed trend represents a genuine concentration-dependent proliferative effect. Although treatment and control groups were balanced at baseline for age, sex, edentulous duration, and BMI, formal covariate-adjusted statistical modelling incorporating these variables, together with diabetic status, was not performed in the primary analysis; the diabetic sub-population was merged into this analysis, and given that diabetes is known to impair keratinocyte function and wound healing in oral tissues (33–35), a stratified or covariate-adjusted analysis of diabetic versus non-diabetic participants is planned to be reported separately.

## Conclusion

This prospective controlled clinical trial provides preliminary evidence that topical Moringa oleifera stem bark oil is associated with a progressive increase in cytological markers of oral mucosal keratinization in edentulous ridges over a four-week period, an effect not observed in a concurrent water-massage control group, and that the formulation is well tolerated *in vitro*, with the MTT assay (three independent experiments, technical triplicate) confirming non-toxicity and showing a descriptive, concentration-dependent trend toward higher HaCaT keratinocyte viability relative to control, a trend that was not evaluated by inferential statistics and should therefore be regarded as exploratory rather than confirmatory. This controlled comparison is, to our knowledge, among the first of its kind for a herbal mucosal conditioning agent in prosthodontics and provides supportive—though not definitive—evidence that the observed cytological change is related to the Moringa formulation rather than a spontaneous temporal or massage-related phenomenon. Given the absence of a vehicle-controlled arm, participant blinding, histological confirmation, formal inferential statistical testing of the *in vitro* data, and clinically meaningful prosthodontic or patient-reported outcomes, these findings should be regarded as preliminary. The formulation shows promising potential as a pre-prosthetic mucosal conditioning approach, and its further evaluation—particularly for medically compromised and geriatric patients—warrants confirmation in larger, multicentre, randomized, placebo (vehicle)-controlled trials with extended follow-up, histological verification, formally powered and statistically analysed *in vitro* assays, and clinically relevant, patient-centred endpoints before any clinical recommendation can be made.

## Data Availability

The datasets presented in this article are not readily available because they contain participant-level clinical information subject to institutional ethics committee restrictions on public sharing. Requests to access the datasets should be directed to the corresponding author, Rajesh Shetty (rajeshshetty@yenepoya.edu.in).
